# Transcriptome and Expression Profiling Analysis of Recalcitrant Tea (*Camellia sinensis* L.) Seeds Sensitive to Dehydration

**DOI:** 10.1155/2018/5963797

**Published:** 2018-06-05

**Authors:** Xiaofang Jin, Dandan Liu, Linlong Ma, Ziming Gong, Dan Cao, Yanli Liu, Yeyun Li, Changjun Jiang

**Affiliations:** ^1^State Key Laboratory of Tea Plant Biology and Utilization, Anhui Agricultural University, Hefei, Anhui 230036, China; ^2^Fruit and Tea Research Institute, Hubei Academy of Agricultural Sciences, Wuhan, Hubei 430064, China

## Abstract

The tea plant (*Camellia sinensis* (L.) O. Kuntze) is an economically important woody perennial nonalcoholic health beverage crop. Tea seeds are categorized as recalcitrant and are sensitive to dehydration treatment. However, the molecular basis of this phenomenon has not been investigated. Thus, we analyzed the genome-wide expression profiles of three dehydration stages using RNA-Seq and digital gene expression (DGE) technologies. We performed de novo assembly and obtained a total of 91,925 nonredundant unigenes, of which 58,472 were extensively annotated. By a hierarchical clustering of differentially expressed genes (DEGs), we found that 8929 DEGs were downregulated and 5875 DEGs were upregulated during dehydration treatment. A series of genes related to ABA biosynthesis and signal transduction, transcription factor, antioxidant enzyme, LEA protein, and proline metabolism that have been reported to function in dehydration process were found to be downregulated. Additionally, the expression profiles of 12 selected genes related to tea seed dehydration treatment were confirmed by qRT-PCR analysis. To our knowledge, this is the first genome-wide study elucidating the possible molecular mechanisms of sensitivity of recalcitrant tea seeds to dehydration. The results obtained in this study contribute to the preservation of tea seeds as genetic resources and can also be used to explore the mechanism of dehydration sensitivity of other recalcitrant seeds.

## 1. Introduction

The tea plant (*Camellia sinensis* (L.) O. Kuntze) is native to southwestern China and is an important economic woody perennial crop that is mainly grown in tropical and subtropical regions [1]. Tea is a widely consumed beverage in the world owing to its health benefits [2]. Currently, tea genetic resources are conventionally preserved in germplasm repositories, but are susceptible to potential losses due to diseases, pests, climatic hazards, and so on. Therefore, it is important to preserve tea genetic resources by seed storage for future experimentation. However, many studies have showed that tea seeds are recalcitrant [3–5].

To develop methods to preserve recalcitrant seeds, it is essential to understand the mechanism of sensitivity of these seeds to dehydration treatment. When recalcitrant seeds sense dehydration stress, a series of changes occur, including resetting of the cellular framework, alteration of the structure, composition and function of the plasma membrane, and accumulation of reactive oxygen species (ROS) [6–10]. Although we have already made some progress in elucidating the mechanism of dehydration sensitivity of some recalcitrant seeds, most of it, especially the molecular mechanism, remains unknown. Meanwhile, recalcitrant seeds of different species display a wide range of variability in sensitivity to dehydration [4, 11].

Only a few studies have been conducted to explore the potential mechanisms contributing to dehydration sensitivity of recalcitrant tea seeds. A previous study showed that when tea seeds were subjected to dehydration treatment, there was a dramatic induction of H_2_O_2_ accumulation in tea embryos. Such high levels of ROS were found to be detrimental to seed viability [7]. Some studies have elucidated the molecular mechanism of the response of the tea plant to dehydration stress and identified a series of dehydration-responsive genes [12, 13]. However, the molecular mechanisms of response to dehydration stress in tea plant are tissue-specific, and little is known about this mechanism in tea seeds, especially at the genome-wide transcriptional level.

Recently, rapid advances in next-generation sequencing technologies have proved to be a cost-effective method for transcriptomic studies [14, 15]. Therefore, the purpose of this study was to identify dehydration-responsive genes and elaborate on the mechanisms of dehydration sensitivity in recalcitrant tea seeds by RNA-Seq and DGE studies. The results obtained in this study not only allow us a deeper insight into the molecular mechanisms of sensitivity of recalcitrant tea seeds to dehydration, but will also be useful for preserving tea genetic resources by seed storage for a long time.

## 2. Materials and Methods

### 2.1. Plant Materials and Seed Dehydration Treatments

Mature seeds with brown pericarp were collected from the tea plant cultivar “*C. sinensis* cv. Echa 1” grown in Wuhan, Central China (114°07′E, 30°18′N). Of these, only healthy seeds were selected for use in our experiments. The pericarps of the seeds were removed and placed in an electrothermal blowing dry box (25°C) to allow dehydration for 0, 1, 3, 5, 8, 11, 14, and 18 d. The moisture contents of the seeds were determined gravimetrically by oven drying at 103°C for 17 h [16]. Three replicates of 30 seeds each were used to determine the seed moisture content measured on a fresh mass basis.

### 2.2. Seed Germination Tests

Seed germination test was carried out in an artificial climate chest (25°C, 500 lx, 16 h light/8 h dark) after placing the seeds in 15 cm diameter petri dishes containing wet filter paper [5, 7]. At each sampling point, three replicates of 50 seeds each were used for the germination test. Germination was defined as the appearance of a radical of at least 5 mm in length [5]. Based on seed germination rates, samples at three stages, that is, fresh seeds (CK, D0) and partially dehydrated (D1 and D2), were selected for RNA-Seq and DGE analyses. Three biological replicates of 50 seed embryos each were quickly frozen in liquid nitrogen and stored at −80°C for RNA extraction.

### 2.3. RNA Extraction

RNAprep pure Plant Kit (Tiangen, Beijing, China) was used for RNA extraction. The concentration, integrity, and purity of RNA were assessed using Agilent 2100 Bioanalyzer (Agilent Technologies, Santa Clara, CA, USA) and NanoDrop ND-2000 spectrophotometer (NanoDrop Technologies, Wilmington, DE, USA). All RNA samples (including a pooled RNA sample for transcriptome sequencing and RNA preparations from D0, D1, and D2 samples for DGE sequencing) with A260/A280 ratios ranging from 1.9 to 2.2 and RNA integrity number (RIN) values greater than 8 were chosen to construct cDNA libraries and quantitative real-time PCR (qRT-PCR) analyses.

### 2.4. Library Construction and Quality Control

Following DNase I treatment, mRNA was enriched by oligo(dT) magnetic beads. The mRNA mixed with fragmentation buffer was sheared into short fragments, which were used as templates for cDNA synthesis. Double-stranded cDNA was purified using magnetic beads and resuspended in EB buffer for end repair and addition of poly-(A) tails. Finally, sequencing adapters were ligated to the fragments, and suitable fragments were selected as templates for PCR amplification. During the quality control step, Agilent 2100 Bioanaylzer was used to check the quality and to quantify the sample libraries. The libraries were sequenced using Illumina HiSeq™ 2500 (Illumina, San Diego, USA) to generate raw data. These data are available at the NCBI sequence read archive (SRA) under the accession number SRP096975. Raw reads were preprocessed to remove reads with adapters, reads containing more than 10% unknown bases, and low-quality reads (>50% of the bases with a quality score of ≤5).

### 2.5. De Novo Assembly and Functional Annotation

The filtered reads were de novo assembled into nonredundant unigenes by the Trinity software [17] and TGICL software [18]. To obtain information on the functional annotation, all unigenes were used to search against the nonredundant database (NR), the nucleotide database (NT), the Swiss-Prot protein sequence database (Swiss-Prot), the cluster of orthologous group database (COG), the Kyoto encyclopedia of genes and genomes database (KEGG) by the BLAST algorithm, and the InterPro protein families database (InterPro) with InterProScan5 (*E* value ≤ 10^−5^). Proteins with highest sequence similarity to given unigenes were retrieved. Based on NR annotations, gene ontology (GO) annotations were assigned to unigenes using Blast2GO software [19].

### 2.6. Protein Coding Region Prediction

To predict protein coding sequences (CDSs), unigenes were first aligned by BLASTx (*E* value ≤ 10^−5^) to protein databases in the priority order of NR, Swiss-Prot, KEGG, and COG. Unigenes aligned to a higher priority database were not aligned to a subsequent lower priority database. The coding region sequences of unigenes were determined based on proteins with highest ranks in the BLAST results. These sequences were then translated into amino acid sequences using the standard codon table. When unigenes could not be found in any of the above databases, ESTScan [20] was used to decipher their nucleotide and amino acid sequences.

### 2.7. Read Mapping to the Reference Transcriptome and Differential Expression Analysis

Clean reads and count number of DGE libraries were assessed and summarized using custom BioPerl scripts, and the reads were mapped back onto the assembled transcriptome generated by RNA-Seq. The expression levels of the unigenes were quantified by RSEM software [21] and calculated as FPKM [22]. Significantly differentially expressed unigenes with false discovery rate (FDR) ≤ 10^−3^, *E* values ≤ 10^−5^, and ∣log2 ratio∣ ≥ 1 were identified among D0, D1, and D2 libraries. The cluster analysis of DEGs was performed by using Cluster 3.0 software [23]. To further clarify the biological functions of DEGs, GO and KEGG pathway enrichment analyses were conducted by the Cytoscape software [24] and KOBAS software [25], respectively.

### 2.8. Heatmap and Transcription Factor Analysis

The heatmap was plotted using OmicShare tools, a free online platform for data analysis (http://www.omic-share.com/tools). Transcription factors (TFs) were identified and classified based on the PlantTFDB 3.0 database [26].

### 2.9. Quantitative Real-Time PCR Validation

To validate the expression profiles observed in RNA-Seq data, 12 significantly differentially expressed genes (DEGs) were selected for qRT-PCR analyses using SYBR Premix Ex Taq™ II Kit (Takara, Dalian, China) in a Bio-Rad CFX96 real-time PCR system (Bio-Rad, CA, USA). Glyceraldehyde-3-phosphate dehydrogenase (GAPDH) gene was used as an internal control. The primers used in qRT-PCR analyses are listed in Table S1. The relative expression values were calculated by using the 2^−ΔΔCt^ method [27]. Three biological replicates were performed for each experiment.

## 3. Results and Discussion

### 3.1. Germination Changes of Tea Seeds during Dehydration Treatment

**Figure 1:**
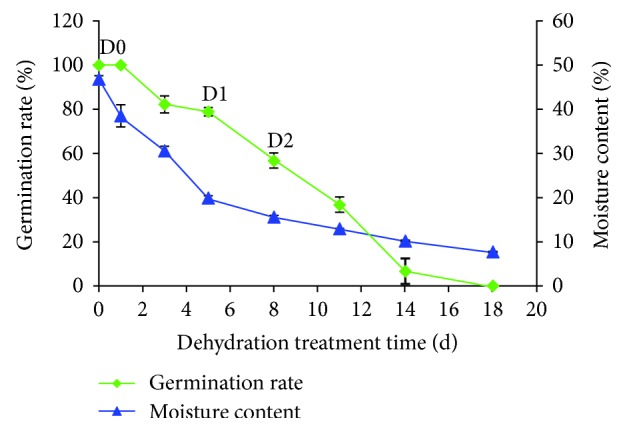
Changes in germination rate and moisture content expressed as fresh weight of recalcitrant tea seeds during dehydration treatment.

In this study, we found that tea seeds at the time of harvest had a moisture content of 46.8% (D0, 0d) and a germination rate of 100% (Figure 1). With continued dehydration, the germination rate of the seeds decreased gradually. When dehydrated to 19.9% (D1, 5d), that is, less than half of the initial moisture content, the germination rate was nearly 80%. However, further dehydration to 15.6% (D2, 8d) sharply reduced the germination rate to 56.7%. When the seeds were further dehydrated, reducing the moisture content to 7.7%, the seeds permanently lost their viability. These results demonstrated that tea seeds are highly sensitive to dehydration treatment, maintaining high viability at 20% moisture content, with a moisture content of about 16% leading to a serious injury. Therefore, to evaluate the transcriptomic response of recalcitrant tea seeds during dehydration treatment, we selected D0, D1, and D2 for RNA-Seq and DGE studies.

### 3.2. Analysis of the Transcriptome

RNAs isolated from D0, D1, and D2 samples were mixed in equal amounts to construct a broad cDNA library using the Illumina HiSeq 2500 genome analyzer. Overviews of sequencing and de novo assembly results (Table S2), a total of 100,628,270 clean reads were obtained. These high-quality trimmed reads were then assembled de novo into 91,925 nonredundant unigenes with an average length of 854 bp and an N50 length of 1480 bp. All unigenes were longer than 300 bp, and 28,176 (30.65%) of them were longer than 1000 bp (Figure S1). A total of 48,214 (52.45%), 51,917 (56.48%), 32,028 (34.84%), 17,055 (18.55%), 33,807 (36.78%), 9535 (10.37%), and 28,088 (30.56%) unigenes had significant hits (*E* value ≤ 10^−5^) in NR, NT, Swiss-Prot, COG, InterPro, GO, and KEGG, respectively. About half of all nonredundant unigenes had significant homology with genes in NR and NT. Of the 91,925 high-quality unique sequences, 48,630 (52.90%) unigenes significantly matched known proteins in at least one of the five databases, and 12,678 (13.79%) unigenes showed similarity to proteins in all five databases (Figure S2). Based on the BLASTx protein database searches described above, we could identify 47,449 unigenes containing CDSs with an average length of 806 bp and an N50 length of 1242 bp. Of the unigenes with CDSs, 29,012 (61.14%) were longer than 500 bp, 15,153 (31.94%) were longer than 1000 bp, and 3810 were longer than 2000 bp (Figure S3). Using the ESTScan program, we were further able to assign another 2851 unigene CDSs that could not be aligned to the above databases.

### 3.3. DGE Analysis among the Three Stages of Dehydration

**Figure 2:**
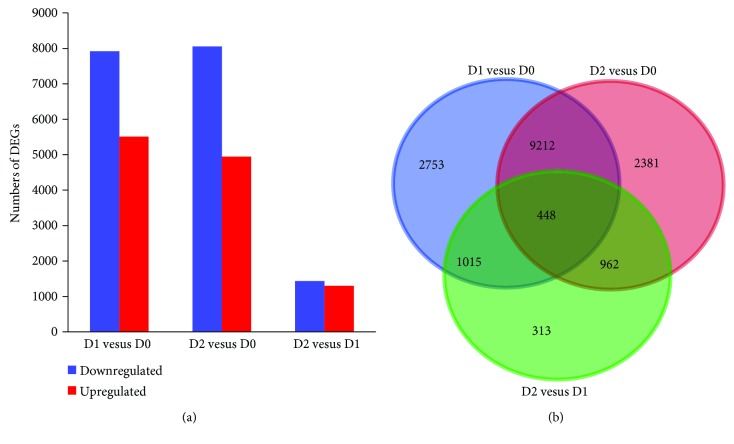
Overview of significantly differentially expressed genes (DEGs) in recalcitrant tea seeds during dehydration treatment. (a) The numbers of downregulated and upregulated DEGs. (b) Venn diagram for analysis of DEGs.

To elucidate the molecular mechanisms of the response of recalcitrant tea seeds to dehydration treatment, DGE analysis was performed to determine DEGs. Approximately 12.9 million clean reads were obtained in each library. Gene annotations were carried out by mapping clean reads to the 91,925 nonredundant unigenes from the transcriptome. About 87.3–88.4% reads in all DGE libraries were mapped to the global transcriptome, suggesting that the transcriptome was a reliable reference. Upon screening of the DEGs, we observed that the level of gene expression changed with the duration of the dehydration treatment process. As shown in the histogram, the proportion of downregulated DEGs was higher than that of the upregulated DEGs among all three dehydration stages (Figure 2(a)). In the D1 versus D0 library, we observed a total of 7917 downregulated DEGs and 5511 upregulated DEGs; in the D2 versus D0 library, we found a total 8056 downregulated DEGs and 4947 upregulated DEGs; in the D2 versus D1 library, we found a total of 1438 downregulated DEGs and 1300 upregulated DEGs. We then made a hierarchical clustering to determine the profiles of the DEGs among the three seed dehydration stages (Figure S4). The results revealed that 482 DEGs were initially downregulated in D1 and were then upregulated in D2, while the opposite trend was observed for 525 DEGs. However, 8929 DEGs and 5875 DEGs were downregulated and upregulated, respectively, in all dehydration treatment stages. Concurrently, by Venn diagram analysis, we found that only 448 DEGs overlapped among the three comparisons (Figure 2(b)).

### 3.4. GO and KEGG Pathway Enrichment Analyses of DEGs

**Figure 3:**
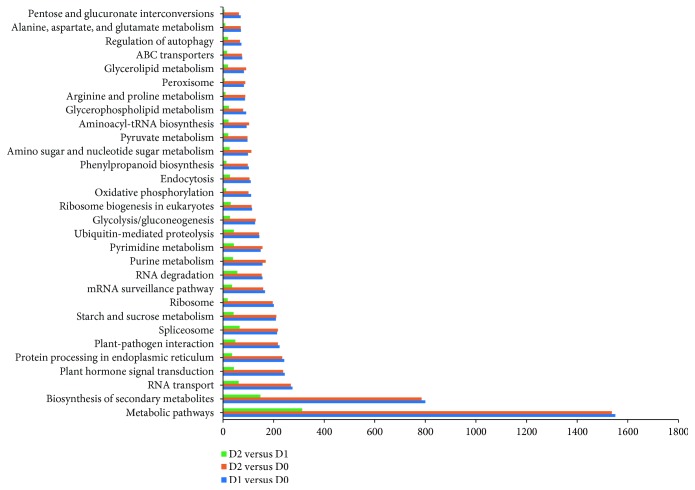
30 main KEGG pathways based on significantly differentially expressed genes (DEGs) in recalcitrant tea seeds during dehydration treatment. The *x*-axis indicates the number of DEGs in each pathway, and the *y*-axis indicates KEGG pathway categories.

Upon GO enrichment analysis, we found that several crucial biological processes, such as carbohydrate metabolic process, amino acid metabolic process, and coenzyme metabolic process, were significantly (*p* ≤ 0.05) enriched (Table S3). With KEGG pathway enrichment analysis [28], 127, 127, and 113 KEGG pathways were identified in D1 versus D0, D2 versus D0, and D2 versus D1 libraries, respectively. Among these KEGG pathways, the largest groups were metabolic pathways, biosynthesis of secondary metabolites, RNA transport, and plant hormone signal transduction (Figure 3). Further, we found that a total of 14, 17, and 7 KEGG pathways were significantly (*p* ≤ 0.05) enriched in D1 versus D0, D2 versus D0, and D2 versus D1 libraries, respectively (Table S4). Several key pathways, such as arginine and proline metabolism (ko00330), fatty acid biosynthesis (ko00061), inositol phosphate metabolism (ko00562), carotenoid biosynthesis (ko00906), and basal transcription factors (ko03022), were involved and function in the response to dehydration treatment of recalcitrant tea seeds. These results imply that genes involved in these biological processes and metabolic pathways may play crucial roles in the dehydration sensitivity of recalcitrant tea seeds.

### 3.5. Abscisic Acid Biosynthesis and Signal Transduction Genes Responding to Seed Dehydration Treatment

**Figure 4:**
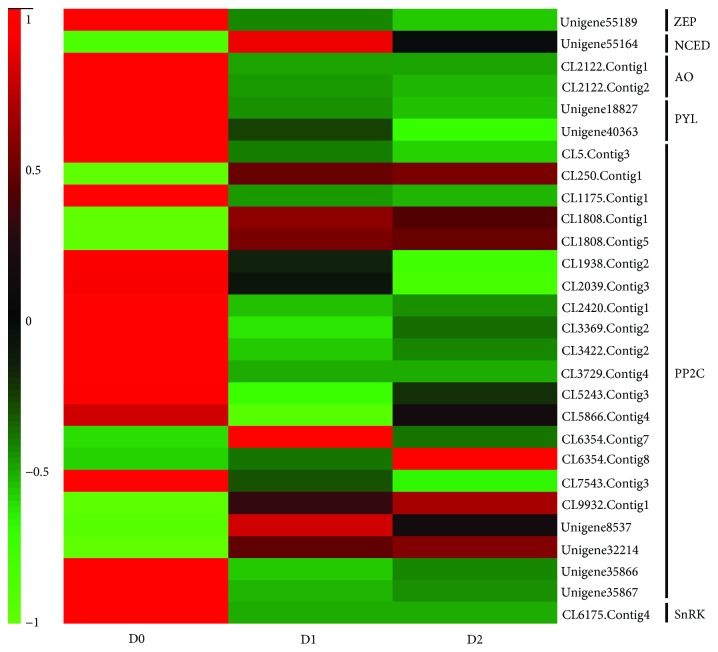
Heatmap representing relative expression levels of differentially expressed genes (DEGs) related to ABA biosynthesis and signal transduction. The numbers in the scale bar show the *Z*-score standardization of each DEG.

Abscisic acid (ABA) is an important hormone that plays vital roles in adaptive responses to various environmental stresses [29] and has been also verified as a vital factor in seed dehydration tolerance [30, 31]. In our study, one gene encoding zeaxanthin epoxidase (ZEP), one gene encoding 9-cis-epoxycarotenoid dioxygenase (NCED), and two genes encoding aldehyde oxidase (AO) related to the ABA biosynthesis pathway were identified to be differentially expressed (Figure 4 and Table S5). Although *NCED* was upregulated, the downregulation of *ZEP* and *AO* might restrain ABA biosynthesis during recalcitrant tea seed dehydration treatment. At the same time, we identified two genes encoding PYR1-like (PYL), one gene encoding SNF1-related protein kinase (SnRK), and 21 genes encoding protein phosphatase 2C (PP2C) that constitute the core regulatory network of ABA signaling pathway (Figure 4 and Table S5) and can activate a series of transcription factors (e.g., bZIP) to cope with dehydration stress [32]. Interestingly, we found that most or all of these three genes (*PYL*, *PP2C*, and *SnRK*) were also downregulated. Thus, we speculate that the repression of genes encoding ABA synthesis and signaling is likely to lead to the dehydration sensitivity of recalcitrant tea seeds.

### 3.6. Transcription Factors Responding to Seed Dehydration Treatment

**Figure 5:**
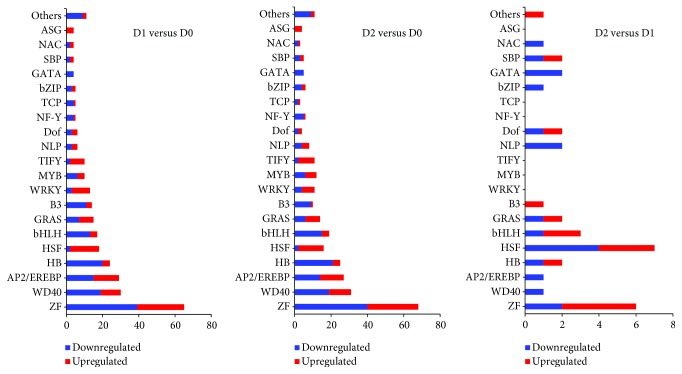
Distribution of differentially expressed TFs in recalcitrant tea seeds during dehydration treatment. The histograms show the number of downregulated and upregulated TFs in D1 versus D0, D2 versus D0, and D2 versus D1 libraries, respectively. Details are not shown for TF gene families with less than four unigenes.

Many studies have demonstrated that transcription factors play important functions in tolerance to abiotic stresses [33–35]. In our study, a total of 299 (171 downregulated and 128 upregulated), 299 (174 downregulated and 125 upregulated), and 34 (19 downregulated and 15 upregulated) genes encoding putative transcription factors were identified in D1 versus D0, D2 versus D0, and D2 versus D1 libraries, respectively (∣log2 ratio∣ ≥ 1), with the number of downregulated TFs being higher. These TFs could be divided into 29 gene families based on their putative DNA-binding and kinase domains (Figure 5); most of the members of the 13 TF gene families (zinc finger protein (ZF), WD40, AP2/EREBP, HB, bHLH, B3, MYB, NLP, NF-Y, TCP, bZIP, GATA, and NAC) were downregulated. Among these, at least six TF gene families have been reported to be linked to positive regulation of the responses to dehydration stress and comprise AP2/EREBP [36], bZIP [37], HB [38], MYB [39], NAC [35], and ZF [40]. Furthermore, it has also been reported that these six TF gene families are involved in drought stress resistance of tea plants [13]. Consequently, it can be inferred that these six TF gene families might play vital roles in the response to dehydration sensitivity of recalcitrant tea seeds. In addition, a number of previously published reports showed that HSF and WRKY gene family members can improve the tolerance to drought stress in tea plants [13, 41, 42]. In the present study, we found most of the members of the HSF and WRKY gene families to be upregulated (Figure 5), and we speculated that the upregulated genes of these two TF gene families might help increase the tolerance to dehydration treatment in recalcitrant tea seeds.

### 3.7. Antioxidant Enzymes and Osmoprotectant Metabolism Genes Responding to Seed Dehydration Treatment

**Figure 6:**
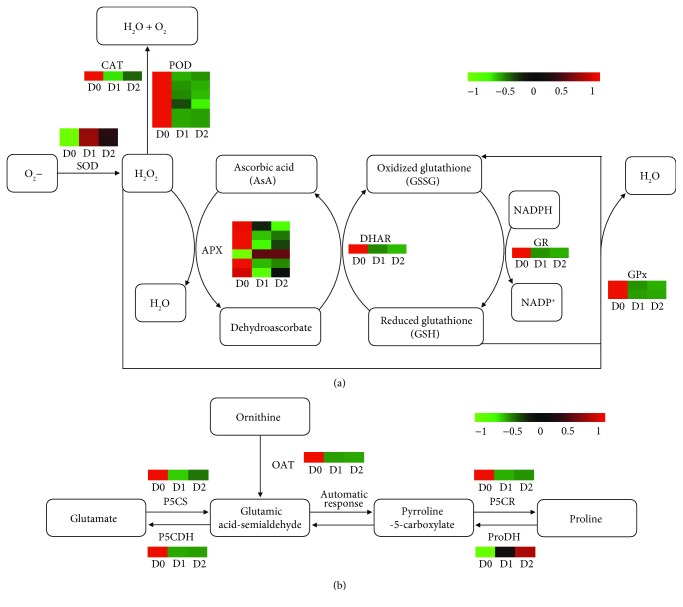
Heatmap representing relative expression levels of differentially expressed genes (DEGs) related to antioxidant enzymes (a) and proline metabolism (b). The numbers in the scale bar show the *Z*-score standardization of each DEG.

During dehydration treatment of recalcitrant seeds, the plasma membrane is believed to be a primary site of injury by ROS (e.g., hydrogen peroxide (H_2_O_2_) and superoxide anion (^•^O_2_^−^)), resulting in loss of viability [16, 43]. Several studies showed that antioxidant system comprising of enzymatic and nonenzymatic (e.g., ascorbic acid (AsA) and glutathione (GSH)) components plays an important role in scavenging excess of ROS [7, 9, 44]. The enzymatic antioxidants include catalase (CAT), glutathione peroxidase (GPx), peroxidase (POD), superoxide dismutase (SOD), and enzymes of AsA-GSH cycle (such as ascorbate peroxidase (APX), dehydroascorbate reductase (DHAR), and glutathione reductase (GR)). In our transcriptome analysis, 2 gene encoding SOD, 1 gene encoding CAT, 6 gene encoding POD, 6 gene encoding APX, 1 gene encoding DHAR, 1 gene encoding GR, and 2 gene encoding GPx were identified (Figure 6(a) and Table S6). Most or all members of the six genes (APX, CAT, DHAR, GPx, GR, and POD) were downregulated, while the opposite trend was observed only for SOD gene. Although all members of *SOD* were upregulated, it only can scavenge ^•^O_2_^−^, thereby resulting in the production of H_2_O_2_ [45, 46]. Therefore, we speculate that the dehydration sensitivity of recalcitrant tea seeds can be related to an accumulation of ROS due to decreased activities of these antioxidant enzymes.

Many previous studies showed that an accumulation of LEA proteins is correlated with gaining seed dehydration tolerance [8, 47]. In our study, all members of LEA proteins were found to be downregulated (Table S6). Therefore, we can speculate that reduced expression of genes encoding LEA proteins results in dehydration-intolerant tea seeds. In addition, it was also reported that accumulation of proline as an osmoprotectant has a positive influence in response to drought stress in tea plant [13]. In this study, one gene encoding pyrroline-5-carboxylate synthetase (P5CS), one gene encoding ornithine aminotransferase (OAT), one gene encoding pyrroline-5-carboxylate reductase (P5CR), one gene encoding proline dehydrogenase (ProDH), and one gene encoding pyrroline-5-carboxylate dehydrogenase (P5CDH) related to proline metabolism were also identified (Figure 6(b) and Table S6). All members of *P5CS*, *OAT*, *P5CR*, and *P5CDH* were downregulated, while those of *ProDH* were upregulated. Therefore, we inferred that proline may not play an important protective role in the response to dehydration treatment of recalcitrant tea seeds.

### 3.8. qRT-PCR Validation of DEGs

**Figure 7:**
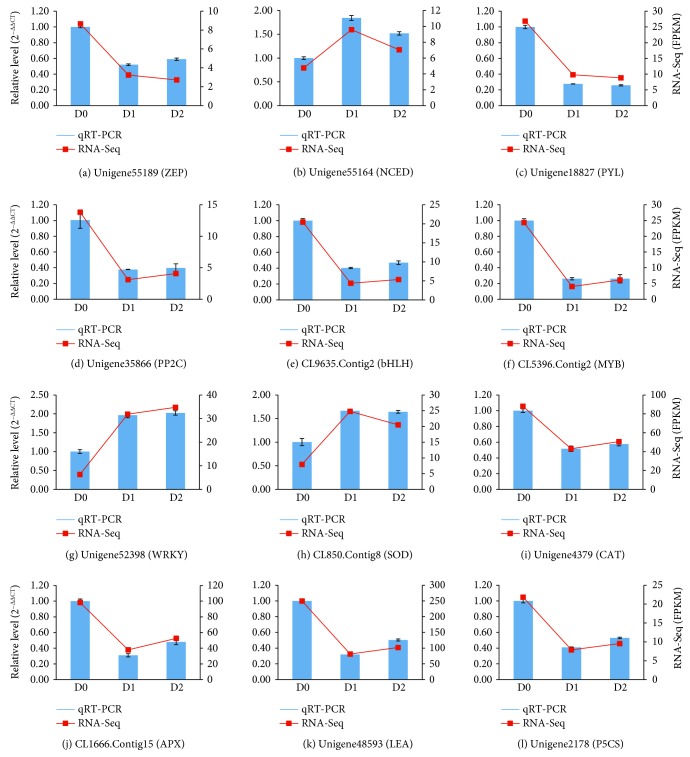
qRT-PCR validation of 12 selected differentially expressed genes (DEGs) responding to tea seed dehydration treatment. Validation of RNA-Seq results using qRT-PCR; GAPDH gene was chosen as the reference gene.

To validate the reliability of our RNA-Seq results, 12 DEGs related to seed dehydration treatment were selected for qRT-PCR analysis. These DEGs are involved in ABA biosynthesis and signal transduction, antioxidant enzyme, LEA protein, and so on. The results of qRT-PCR revealed that the expression trends of each of these DEGs were similar to that from the RNA-Seq results (*R* ^2^ = 0.85). Detailed comparisons between qRT-PCR and RNA-Seq results are shown in Figure 7.

## 4. Conclusion

In conclusion, to the best of our knowledge, this study is the first exploration of the global transcriptome profiles of dehydration sensitivity of recalcitrant tea seeds using RNA-Seq and DGE technologies. In our study, a total of 91,925 nonredundant unigenes were generated by de novo assembly and were extensively annotated, which were then used as the reference database for identification of DEGs. Many candidate DEGs involved in the response to dehydration treatment in recalcitrant tea seeds were identified. A series of genes related to ABA biosynthesis and signal transduction, antioxidant enzyme, LEA protein, and so on reported to function in dehydration process were downregulated, and these genes might play vital roles in the dehydration sensitivity of recalcitrant tea seeds. Therefore, our study provides insights into the molecular mechanisms of dehydration sensitivity in recalcitrant tea seeds. This knowledge is not only useful for the preservation of seeds as tea genetic resources for a long time, but also can be used to explore the mechanism of sensitivity to dehydration of other recalcitrant seeds.

## Data Availability

The data used to support the findings of this study are included within the article, the supplementary materials, or are available upon request.

## Supplementary Materials

Supplementary 1 Figure S1: length distribution of unigenes.

Supplementary 2 Figure S2: venn diagram showing a search results against five databases.

Supplementary 3 Figure S3: CDSs predicted by BLASTx and ESTScan.

Supplementary 4 Figure S4: heatmap of differentially expressed genes (DEGs).

Supplementary 5 Table S1: primers used for qRT-PCR analysis of 12 genes.

Supplementary 6 Table S2: overviews of sequencing and de novo assembly.

Supplementary 7 Table S3: significantly enriched GO terms of DEGs.

Supplementary 8 Table S4: significantly enriched KEGG pathways of DEGs.

Supplementary 9 Table S5: DEGs related to ABA biosynthesis and signal transduction.

Supplementary 10 Table S6: DEGs related to antioxidant enzymes and osmoprotectant metabolism.
