# Supplementary figures and images for: Transcriptome and Expression Profiling Analysis of Recalcitrant Tea (Camellia sinensis L.) Seeds Sensitive to Dehydration
Source: Int J Genomics. 2018 Jun 5;2018:5963797. doi: 10.1155/2018/5963797 (PMC6008840; doi:10.1155/2018/5963797)

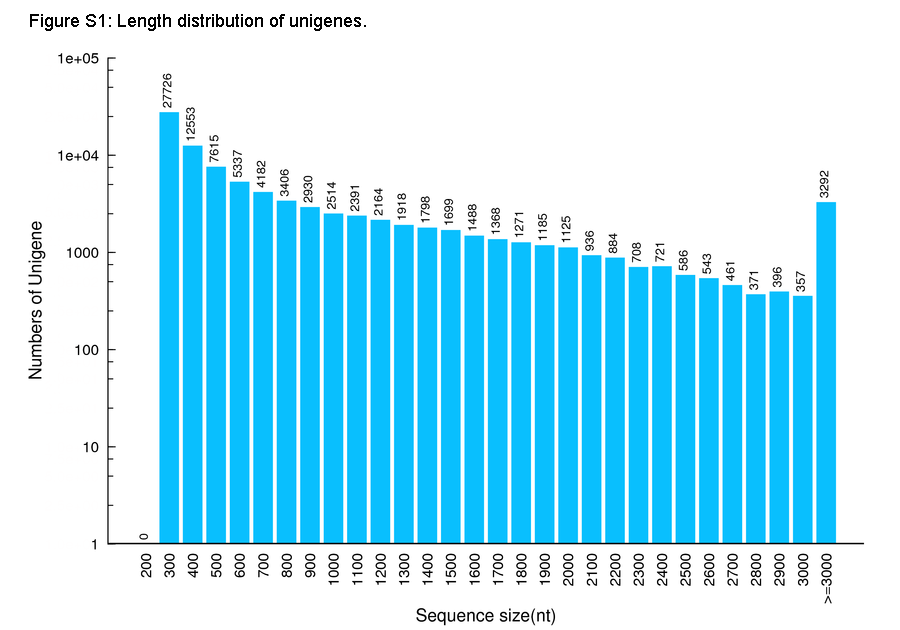

Supplement: Supplementary 1 — Figure S1: length distribution of unigenes. [file 5963797.f1.png]

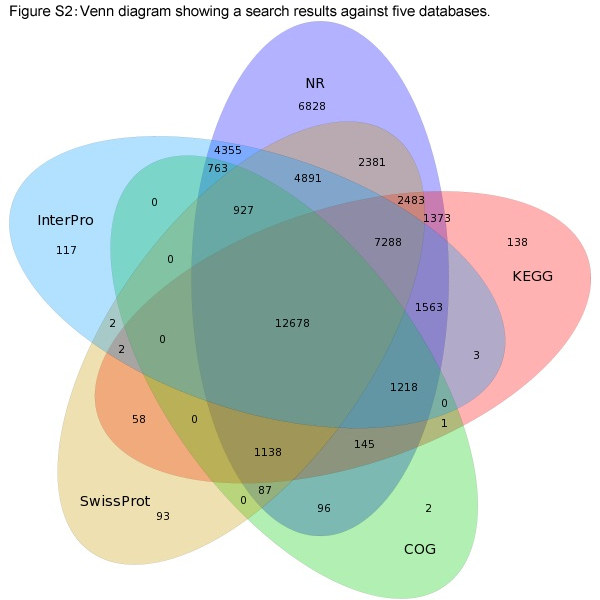

Supplement: Supplementary 2 — Figure S2: venn diagram showing a search results against five databases. [file 5963797.f2.png]

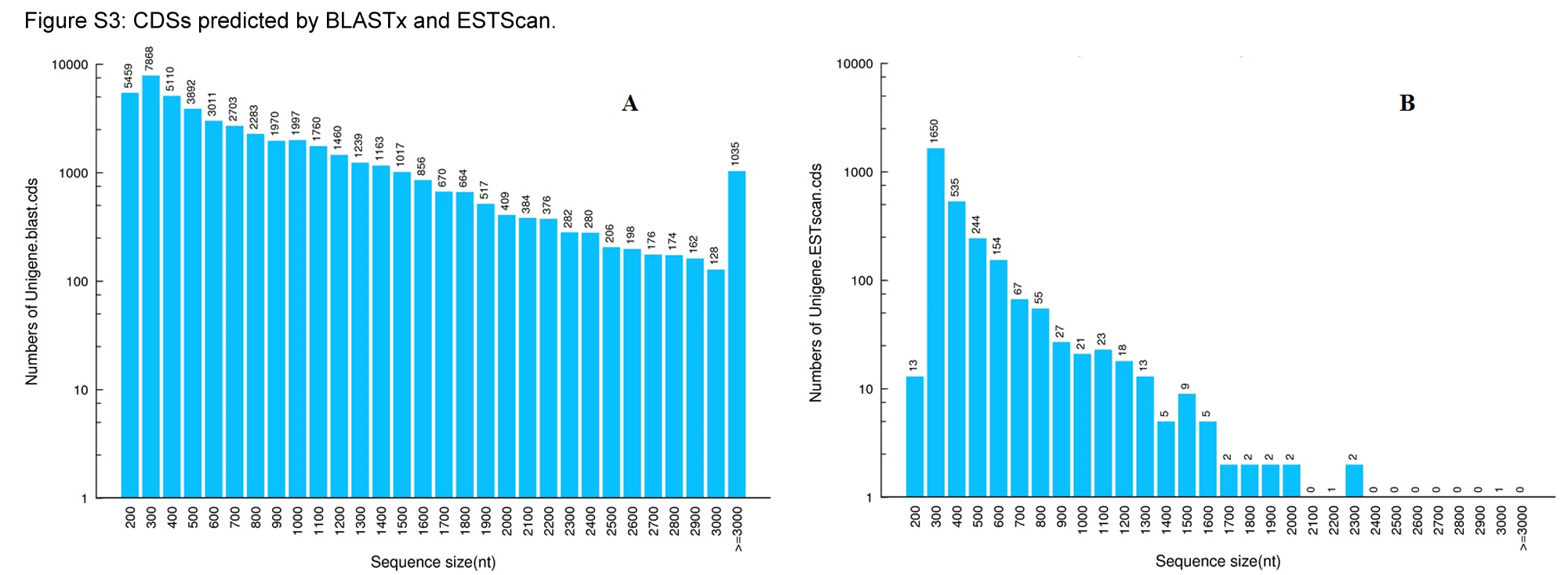

Supplement: Supplementary 3 — Figure S3: CDSs predicted by BLASTx and ESTScan. [file 5963797.f3.png]

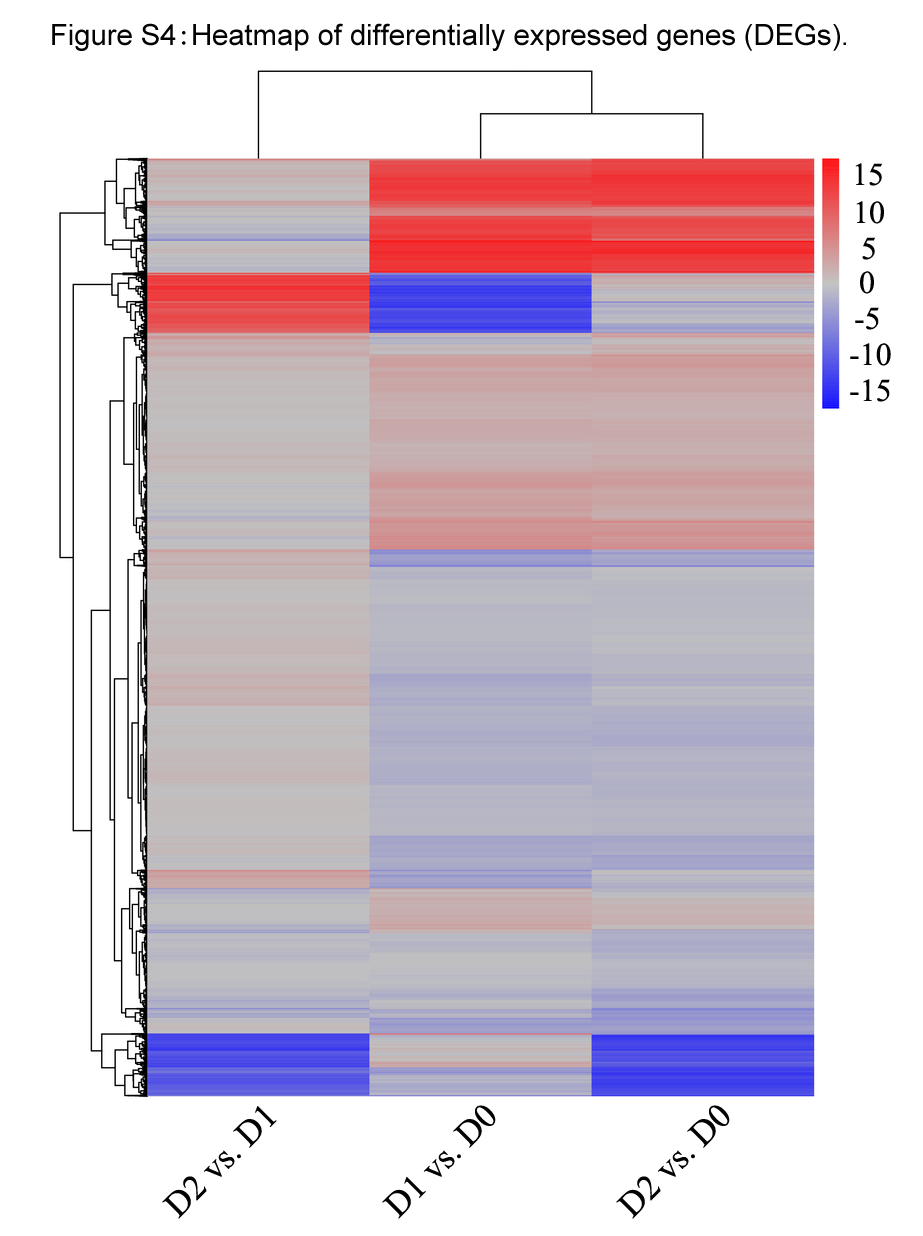

Supplement: Supplementary 4 — Figure S4: heatmap of differentially expressed genes (DEGs). [file 5963797.f4.png]
