# Supplementary material for: Transcriptome and Expression Profiling Analysis of Recalcitrant Tea (Camellia sinensis L.) Seeds Sensitive to Dehydration
Source: Int J Genomics. 2018 Jun 5;2018:5963797. doi: 10.1155/2018/5963797 (PMC6008840; doi:10.1155/2018/5963797)
Supplement: Supplementary 5 — Table S1: primers used for qRT-PCR analysis of 12 genes. [file 5963797.f5.docx]

Table S1：Primers used for qRT-PCR analysis of 12 genes.

| **Number** | **Gene ID** | **Primer sequence** |
| --- | --- | --- |
| 0 | GAPDH | F: TTGGCATCGTTGAGGGTCT |
|  |  | R: CAGTGGGAACACGGAAAGC |
| 1 | Unigene 55189 (ZEP) | F: GATTTGCGGAGCGAACATGG |
|  |  | R: AGTTGGAAGGCACCCGATAC |
| 2 | Unigene55164 (NCED) | F: TCTGAGAAATGGAGCCAACC |
|  |  | R: GGCAAGAATAGCTGGCTTTG |
| 3 | Unigene18827 (PYL) | F: GCACATCCACCACAAATGAC |
|  |  | R: ACACATCCTTGGCATCAGAA |
| 4 | Unigene35866 (PP2C) | F: CAATTCTTTGGCGTTCATCA |
|  |  | R: ATCGGCTTCTTGTTGCAAAT |
| 5 | CL9635.Contig2 (bHLH) | F: ACGACGGAGGAAGATTGTTG |
|  |  | R: CCGAGTGTTGCCATTTCAG |
| 6 | CL5396.Contig2 (MYB) | F: TTCCTTGGTTTCTGCGACA |
|  |  | R: TGGTGTTTGCTCCATCTTCA |
| 7 | Unigene52398 (WRKY) | F: CTTGGCCTCACAGAGGAAAC |
|  |  | R: CGGGAATCACTACCCAAGAA |
| 8 | CL850.Contig8 (SOD) | F: GACAACAGCCCTCCCAATAA |
|  |  | R: TGCTCCTGAAGATGAGATTCG |
| 9 | Unigene4379 (CAT) | F: CAATGAGGAGTTATTGTTCCACAC |
|  |  | R: TCTCATCTGCCGCTATGGAT |
| 10 | CL1666.Contig15 (APX) | F: AGGGTATGGCCACCAGATAGA |
|  |  | R: GATGCTACCAAGGGAACGGA |
| 11 | Unigene48593 (LEA) | F: CTCCACTTCCACTCACAGCA |
|  |  | R: ATGGCTCGTTCTCTCTCCAA |
| 12 | Unigene2178 (P5CS) | F: TGCTGGCAAACATGGAAGAG |
|  |  | R: AGGAGAACACCCAAGGGAGAT |
