# Supplementary material for: Transcriptome and Expression Profiling Analysis of Recalcitrant Tea (Camellia sinensis L.) Seeds Sensitive to Dehydration
Source: Int J Genomics. 2018 Jun 5;2018:5963797. doi: 10.1155/2018/5963797 (PMC6008840; doi:10.1155/2018/5963797)
Supplement: Supplementary 6 — Table S2: overviews of sequencing and de novo assembly. [file 5963797.f6.docx]

**Table S2: Overviews of sequencing and *de novo* assembly.**

| Statistics of data production | Number |
| --- | --- |
| Total raw reads | 100,922,326 |
| Total clean reads | 100,628,270 |
| Total clean bases (bp) | 9,056,544,300 |
| Q20 (%) | 96.72 |
| N (%) | 0.00 |
| GC (%) | 44.69 |
| Total number of unigenes | 91,925 |
| Average length of unigenes (bp) | 854 |
| N50 of unigenes (bp) | 1,480 |
| N90 of unigenes (bp) | 323 |
