# Supplementary material for: Transcriptome and Expression Profiling Analysis of Recalcitrant Tea (Camellia sinensis L.) Seeds Sensitive to Dehydration
Source: Int J Genomics. 2018 Jun 5;2018:5963797. doi: 10.1155/2018/5963797 (PMC6008840; doi:10.1155/2018/5963797)
Supplement: Supplementary 7 — Table S3: significantly enriched GO terms of DEGs. [file 5963797.f7.docx]

**Table S3：Significantly enriched GO terms of DEGs.**

| **#** | **GO term** | **Cluster frequency** | **Corrected P-value** |
| --- | --- | --- | --- |
| **D1 vs. D0** | | | |
| 1 | carbohydrate metabolic process | 391 (8.2%) | 4.06E-02 |
| **D2 vs. D0** | | | |
| 1 | cellular amino acid metabolic process | 261 (5.6%) | 7.39E-05 |
| 2 | alpha-amino acid metabolic process | 140 (3.0%) | 2.30E-04 |
| 3 | glutamine family amino acid metabolic process | 38 (0.8%) | 1.66E-03 |
| 4 | carbohydrate metabolic process | 394 (8.4%) | 2.32E-03 |
| 5 | coenzyme metabolic process | 112(2.4%) | 1.60E-02 |
| **D2 vs. D1** | | | |
| 1 | purine-containing compound transmembrane transport | 6 (0.6%) | 1.49E-03 |
