# Supplementary material for: Transcriptome and Expression Profiling Analysis of Recalcitrant Tea (Camellia sinensis L.) Seeds Sensitive to Dehydration
Source: Int J Genomics. 2018 Jun 5;2018:5963797. doi: 10.1155/2018/5963797 (PMC6008840; doi:10.1155/2018/5963797)
Supplement: Supplementary 8 — Table S4: significantly enriched KEGG pathways of DEGs. [file 5963797.f8.docx]

**Table S4：Significantly enriched KEGG pathways of DEGs.**

| **#** | **Pathway ID** | **Enriched pathways** | **DEGs with pathway annotation (8488)** | **P-value** |
| --- | --- | --- | --- | --- |
| **D1 vs. D0** | | | | |
| 1 | ko03010 | Ribosome | 201 (2.37%) | 1.13E-05 |
| 2 | ko00941 | Flavonoid biosynthesis | 69 (0.81%) | 2.03E-04 |
| 3 | ko00020 | Citrate cycle (TCA cycle) | 55 (0.65%) | 2.86E-03 |
| 4 | ko03020 | RNA polymerase | 56 (0.66%) | 3.22E-03 |
| 5 | ko00196 | Photosynthesis - antenna proteins | 7 (0.08%) | 9.03E-03 |
| 6 | ko03060 | Protein export | 47 (0.55%) | 9.03E-03 |
| 7 | ko00330 | Arginine and proline metabolism | 87 (1.02%) | 1.66E-02 |
| 8 | ko00670 | One carbon pool by folate | 26 (0.31%) | 1.95E-02 |
| 9 | ko00061 | Fatty acid biosynthesis | 37 (0.44%) | 1.97E-02 |
| 10 | ko03050 | Proteasome | 52 (0.61%) | 2.11E-02 |
| 11 | ko04141 | Protein processing in endoplasmic reticulum | 242 (2.85%) | 2.37E-02 |
| 12 | ko00510 | N-Glycan biosynthesis | 63 (0.74%) | 2.67E-02 |
| 13 | ko00900 | Terpenoid backbone biosynthesis | 58 (0.68%) | 3.09E-02 |
| 14 | ko00053 | Ascorbate and aldarate metabolism | 36 (0.42%) | 4.24E-02 |
| **D2 vs. D0** | | | | |
| 1 | ko03010 | Ribosome | 197 (2.35%) | 2.68E-05 |
| 2 | ko00941 | Flavonoid biosynthesis | 70 (0.83%) | 7.73E-05 |
| 3 | ko03020 | RNA polymerase | 58 (0.69%) | 8.17E-04 |
| 4 | ko04122 | Sulfur relay system | 21 (0.25%) | 1.71E-03 |
| 5 | ko04712 | Circadian rhythm - plant | 70 (0.83%) | 1.99E-03 |
| 6 | ko00061 | Fatty acid biosynthesis | 39 (0.46%) | 5.46E-03 |
| 7 | ko00020 | Citrate cycle (TCA cycle) | 53 (0.63%) | 6.30E-03 |
| 8 | ko00330 | Arginine and proline metabolism | 88 (1.05%) | 8.91E-03 |
| 9 | ko00240 | Pyrimidine metabolism | 156 (1.86%) | 1.17E-02 |
| 10 | ko00970 | Aminoacyl-tRNA biosynthesis | 103 (1.23%) | 1.69E-02 |
| 11 | ko00230 | Purine metabolism | 169 (2.01%) | 2.34E-02 |
| 12 | ko00906 | Carotenoid biosynthesis | 35 (0.42%) | 2.93E-02 |
| 13 | ko00196 | Photosynthesis - antenna proteins | 6 (0.07%) | 3.76E-02 |
| 14 | ko00620 | Pyruvate metabolism | 97 (1.16%) | 3.91E-02 |
| 15 | ko00750 | Vitamin B6 metabolism | 13 (0.15%) | 4.36E-02 |
| 16 | ko03060 | Protein export | 43 (0.51%) | 4.59E-02 |
| 17 | ko00260 | Glycine, serine and threonine metabolism | 68 (0.81%) | 4.80E-02 |
| **D2 vs. D1** | | | | |
| 1 | ko00510 | N-Glycan biosynthesis | 21 (1.09%) | 5.62E-03 |
| 2 | ko03018 | RNA degradation | 57 (2.95%) | 1.05E-02 |
| 3 | ko03022 | Basal transcription factors | 16 (0.83%) | 1.41E-02 |
| 4 | ko00240 | Pyrimidine metabolism | 43 (2.23%) | 1.53E-02 |
| 5 | ko00562 | Inositol phosphate metabolism | 23 (1.19%) | 3.13E-02 |
| 6 | ko03040 | Spliceosome | 66 (3.42%) | 3.60E-02 |
| 7 | ko04120 | Ubiquitin mediated proteolysis | 43 (2.23%) | 4.53E-02 |
